# Machine learning for prompt estimation of macroseismic intensity from seismometric data in Italy

**DOI:** 10.1038/s41598-026-35740-x

**Published:** 2026-02-04

**Authors:** Luca Patelli, Michela Cameletti, Valerio De Rubeis, Nicola Alessandro Pino, Claudia Piromallo, Paola Sbarra, Patrizia Tosi

**Affiliations:** 1 Department of Economics, University of Bergamo, Via dei Caniana 2, 24127 Bergamo, Italy; 2 Istituto Nazionale di Geofisica e Vulcanologia (INGV), Via di Vigna Murata 605, 00143 Roma, Italy; 3 School of Science and Technology, Geology Section, University of Camerino, Via Gentile III Da Varano 7, 62032 Camerino, Italy

**Keywords:** Earthquake, Random Forest, Surrogate tree, Macroseismic intensity, Explainability, Uncertainty quantification, Mathematics and computing, Natural hazards, Solid Earth sciences

## Abstract

After an earthquake, it is crucial to rapidly and accurately estimate macroseismic intensity to guide rescue operations and assess potential damage. The Mercalli-Cancani-Sieberg intensity scale is used to qualitatively assess the ground shaking based on observed effects. This study develops a Machine Learning framework, leveraging the Random Forest algorithm, to estimate macroseismic intensity using early available seismic data. Data from different sources are used for model training: seismic data from the Italian instrumental monitoring networks of *Istituto Nazionale di Geofisica e Vulcanologia* and *Protezione Civile*, as well as macroseismic intensity data from both the online macroseismic questionnaire and the on-site surveys by field experts. In order to explain the predictive mechanism of the Random Forest algorithm, this study makes use of surrogate decision trees, providing an interpretative key for the informed decision-making process during seismic events. These models provide insights into the relationships between covariates and predicted intensities, enabling the discussion of model complexity, predictive capability, and explainability. Furthermore, the uncertainty associated with the predictions of the surrogate trees is assessed. When compared with other models for estimating intensity based on ground motion peaks or source parameters, the Random Forest model achieved better predictive performance.

**Supplementary Information:**

The online version contains supplementary material available at 10.1038/s41598-026-35740-x.

## Introduction

Earthquakes, especially those of the highest magnitude, pose a major global risk due to their sudden onset and limited predictability, which can lead to extensive damage and loss of life without prior warning. This inherent unpredictability complicates the development and deployment of reliable alert systems and organized first-response efforts^1^. Historical records of seismic events illustrate that prompt and accurate estimation of the effects of earthquakes can substantially improve emergency response, helping to save lives and reduce economic losses^2^. A key tool for this is macroseismology, the study of the effects of earthquakes on people, objects and buildings. This approach is not only fundamental for understanding past seismic events, but also an essential instrument for assessing the impact of current earthquakes, especially in areas with limited seismological instrumentation.

Macroseismic intensity, commonly measured in Italy via the Mercalli-Cancani-Sieberg (MCS) scale^3^, is based on reports of earthquake effects from either voluntary online observers or expert on-site surveys. The time it takes to estimate intensity varies significantly, from hours for web-based data to several months for expert surveys. Given that rapid intensity estimates are crucial for guiding rescue operations and damage assessment, the seismological community has developed methods for prompt estimation. More recently, non-traditional data sources, from social media, like Twitter have also been explored for rapid intensity estimation^4, 5^; however, the common approach based on seismological data involves using empirical laws like Intensity Prediction Equations (IPEs), based on source location and magnitude, or Ground Motion to Intensity Conversion Equations (GMICEs), which convert instrumental data to intensity, for short-term estimations. While numerous equations for this purpose exist (e.g., Gomez-Capera et al.^6^; Zanini et al.^7^), they often focus on a direct relationship with either earthquake source variables (distance and magnitude) or ground motion measurements. However, a study by Worden et al.^8^ demonstrated that combining both types of data significantly improves the accuracy of the estimates, highlighting the benefit of a hybrid model.

Machine Learning algorithms represent an innovative alternative for prompt intensity estimation. They have already been successfully applied across various domains, such as finance, healthcare, and environmental science, where they have demonstrated a powerful ability to analyze complex phenomena and detect subtle patterns in the data that traditional methods might overlook^9^. By identifying possible non-linear relationships among seismic variables, Machine Learning could enhance predictive accuracy providing timely and reliable intensity estimates.

The use of Machine Learning for seismological problems has increased in recent years (e.g., Beroza et al.^10^), also for the prediction of seismic intensity. Common approaches use earthquake characteristics like magnitude, focal depth, and epicentral distance^11–15^, along with amplitude and energy parameters^16^. Other papers utilize local seismic data such as waveforms and ground motion variables from monitoring stations for on-site intensity predictions^17, 18^. The field has also expanded to integrate a wider variety of data sources. Some studies have incorporated telecommunication data^19^ and georeferenced social media contents^20, 21^, enriching the dataset available for training. Furthermore, Machine Learning models have been explored using accelerograph data from smartphones, with predictors like shaking duration and peak ground acceleration^22^. A crucial and growing consideration for the adoption of these models is the explainability of their predictions^16, 23^. Providing decision-makers and experts with an interpretable methodology is essential for building trust and ensuring the effective use of these advanced systems.

In this work, we propose a framework for the prompt estimation of macroseismic intensity in Italy, i.e., a few minutes after the occurrence of a seismic event. The core of this framework is a Random Forest (RF) algorithm trained to predict intensity values. To address the inherent “black-box” nature of RF models, we adopt surrogate trees to approximate the predictive mechanism of the RF. This approach allows us to represent the complex model graphically, providing a clear and interpretable methodology that supports the decision-making process for experts and authorities. Through this model shift, the surrogate trees can be used to directly predict macroseismic intensity. Additionally, we employ impurity measures to quantify the uncertainty associated with these predictions, providing a measure of confidence for each estimate. The predictive performance of the proposed framework is then assessed by comparing it against available empirical models, specifically IPEs and GMICEs. This is conducted through a validation-test evaluation (including multiple earthquakes) and an out-of-sample single-earthquake case study to demonstrate its performance in a real-world scenario.

The paper is organized as follows: the Materials and Methods section details the data selection and processing, describes the models adopted for the predictions, and explains the measures selected to evaluate the predictive performance and uncertainty. The results for RF, surrogate trees, IPEs and GMICEs for both the multi-event dataset and the out-of-sample single-earthquake case are presented in the Results and discussion section. Finally, in the Conclusion section the advantages and the limitations of the proposed framework are discussed.

## Materials and methods

### Data selection and processing

The dataset utilized in this study consists of 5466 observations collected in Italy from 523 distinct seismic events that occurred from 2008 to 2020. Each observation is a statistical unit defined as the area within a 5 km radius of a monitoring station that was triggered by seismic waves. For each of these observations, we have two main categories of variables: macroseismic intensity and seismometric measures (i.e., earthquake source parameters and ground motion parameters).

The macroseismic intensity at a given location measures the severity of the effects of the earthquake on buildings, objects and people. This study utilizes data from two primary Italian sources: field surveys and web-based surveys. Data from field surveys, which refer only to earthquakes that have caused damage, are sourced from the Database Macrosismico Italiano (DBMI; emidius.mi.ingv.it/DBMI)^24^, while web-based surveys are collected via the “Hai Sentito Il Terremoto” database (HSIT, https://www.hsit.it)^25–27^. HSIT provides reliable intensity data^28^ directly from individuals who experienced the earthquake, and has been storing data on the effects of earthquakes since 2007. Macroseismic intensity is assigned to an area of territory that can vary in size. In the DBMI’s direct survey, damage estimates are conducted in localities, while the HSIT intensities considered in this study are assigned to municipalities, which are usually larger in size. In some cases, there may also be a difference in vulnerability between the historical center and the suburbs. For example, in the case of L’Aquila in the earthquake of April 6, 2009 (*M*_*w*_ 6.1), the direct survey of the historical center assigned an intensity of 8–9 MCS, while for the entire municipality, the HSIT estimated intensity was 6 MCS. To overcome the problem arising from the difference, sometimes considerable^29–31^, in the intensities estimated using these two geographic scales, we averaged all intensities of localities and municipalities falling within a 5 km radius of the monitoring station. This choice ensures better representation of the impact of the earthquake on the area surrounding the station and a lower incidence of highly localized site effects due to particular lithologies. These averaged intensities are then rounded to the nearest integer to align with the qualitative ordinal MCS scale. The final dataset combines these sources to ensure a comprehensive representation of intensity values. For events from 2010 to 2020, intensities were sourced from both HSIT (for municipalities with at least three completed questionnaires), and DBMI. In addition, DBMI data from three earthquakes that occurred in 2008–2009 were included, while intensities greater than or equal to 6 assessed for the earthquake of 30 October 2016, *M*_*w*_ 6.5, were excluded to avoid problems due to the cumulative effect of damage^32^. In particular, the DBMI data, which refer to specific localities, may include multiple intensity values for a single area, contributing to the final average. By combining the two types of data in this study, both the reliable high intensity values estimated on site by the DBMI and the numerous low and medium intensity values from the HSIT will be represented in the reference dataset.

**Fig. 1:**
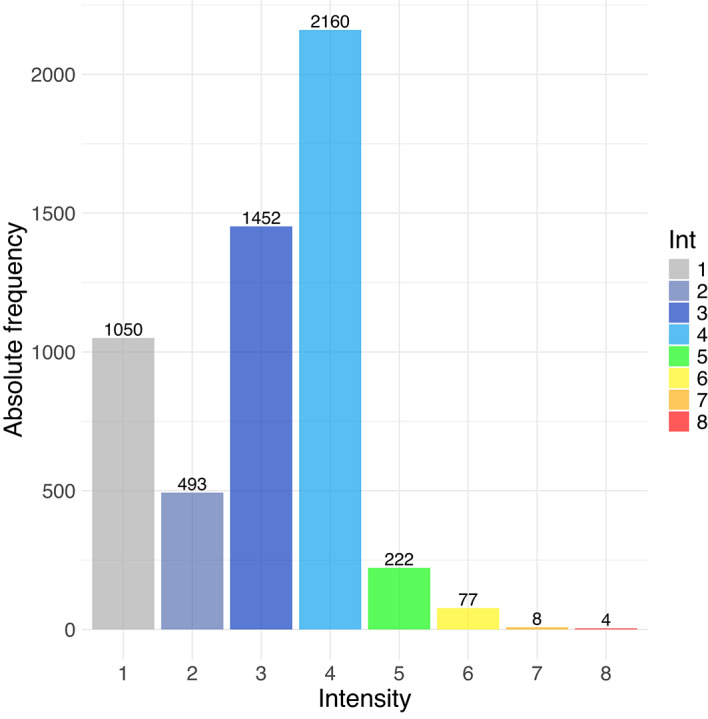
Intensity class distribution in the dataset of this study (combining HSIT and DBMI data).

Figure 1 illustrates the distribution of observed macroseismic intensity classes (intensities from 1 to 8) within the dataset. The maximum intensity class corresponds to 8, as the highest values recorded in DBMI locations (maximum = 10 MCS) were averaged within a 5 km radius around the stations to obtain a value representative of the entire area. The number of data points naturally increases as intensity decreases because seismic waves attenuate with distance from the epicenter, while the spatial range enlarges, and lower-magnitude earthquakes occur more frequently than larger ones. As expected, Fig. 1 shows that lower intensity levels (intensity 4 and below) appear more frequently in the dataset. However, there is a notable underrepresentation of data for the very lowest intensities (intensities 1–3). This is likely due to two factors: a decrease in both seismometric recordings and the number of people who perceive the effects of very small ground shaking^33^. We are aware of the inherent problem of underrepresentation of high intensities in macroseismic databases, which can lead to limitations in estimation.

The first set of seismometric measures is represented by seismic source and propagation parameters, including the geographical coordinates and focal depth (from which the distance to the seismic station is calculated) and the magnitude *M*_*w*_, available from the ITACAext 2.0 dataset^34^. In detail, the considered variables are:

- Mw_trasf: the moment magnitude (*M*_*w*_) which is either calculated directly or estimated using empirical relations based on other magnitude types^35^;
- focal_depth: the earthquake focal depth (in *km*);
- logst_IPODIST: the decimal logarithm of distance (in *km*) between the monitoring station and the hypocenter.

We retained the moment magnitude *M*_*w*_ as a predictor because it is a standard international measure of earthquake size, is readily available in the ITACAext 2.0 dataset^34^, and represents the assigned value for the large majority of our data. Furthermore, extensive literature exists detailing reliable conversion factors for deriving *M*_*w*_ from other magnitude types, ensuring data homogeneity. However, the use of magnitude *M*_*w*_ as a predictor requires some considerations. While *M*_*w*_ is the international standard derived from the seismic moment *M*_*0*_^36^, its definition is known to have limitations. Specifically, it may fail to accurately represent the radiated energy for earthquakes below approximately *M*_*w*_ = 7.5^37^. These small-to-moderate magnitude events dominate the Italian dataset, hence the use of *M*_*w*_ could potentially underestimate the energy released. To address this concern, we also considered the M_wg_^38^ scale, developed to mitigate these limitations.

The second set of seismometric variables consists of instrumental ground motion measures sourced from the ITACAext 2.0 dataset^34^. This includes data from permanent and temporary networks, with a total of more than 1,200 stations. For each metric, we calculated the decimal logarithm of the geometric mean of the maximum values recorded on the two horizontal components of a seismograph. This approach accounts for the fact that the peak shaking on each component may occur at different times during the seismic event. The specific variables considered are:

- GeoMean_pga: decimal logarithm of peak ground acceleration (PGA, in cm/s^2^)
- GeoMean_pgv: decimal logarithm of peak ground velocity (PGV, in cm/s);
- GeoMean_pgd: decimal logarithm of peak ground displacement (PGD, in cm).

We selected these three variables from all the available ground motion measures, such as energy parameters and spectral acceleration ordinates. This was done following preliminary RF testing which showed that there was little variation in the results and slightly higher accuracy for those models which used only this subset of variables.

In general, the whole set of variables could be enriched and/or edited based on the region of application and on the dataset available. For example, different intensity scales, such as the European Macroseismic Scale^39^ or the MMI scale^40^.

### Random forest and surrogate tree

The estimation of earthquake intensity can be seen as a classification problem given the intrinsic qualitative and ordinal nature of the MCS scale. For the *i*-th observation we have the response variable ${y}_{i}\in \{1,\dots ,K\},$ where $K=8$ represents the number of observed intensity classes (categories), and a $P$-dimensional predictor vector ${x}_{i} = {\left({x}_{1},\dots ,{x}_{P}\right)}^{T}$. In our application $P=6$ corresponding to the six seismometric variables described above. Given our dataset denoted by ${D}_{n}={\left\{{y}_{i},{x}_{i}\right\}}_{i=1}^{n}$ we assume the following model:

1 $${y}_{i}=f\left({x}_{i}\right)\space i=1,\dots ,n$$

where $f(\cdot )$ is a function of the $P$ predictors that needs to be estimated by our Machine Learning algorithm.

In order to estimate $f(\cdot )$ (Eq. 1), to be used later for predictive purposes, we adopt the RF algorithm^41^. RF is a non-parametric and flexible model which is able to estimate complex non-linear relationships between predictors and response, including interactions between predictors, without the need to specify a functional form a priori. RF is an ensemble method that aggregates predictions from a large number, *B*, of individual decision trees^42^. Each tree is grown on a bootstrap sample of the original data, obtained by randomly sampling *n* observations from ${D}_{n}$ with replacement. To minimize correlation among the trees, RF introduces an additional level of randomness at each split: the potential candidate predictor is chosen from a random subset of predictors of size $m < P$. This combination of bootstrapping and feature randomness results in a more robust model, with respect to a single decision tree, with a generally higher predictive accuracy. The RF algorithm can be applied to both regression and classification problems, including the multiclass case considered in this paper. Due to the iterative nature of its building blocks (decision trees), RF makes it possible to evaluate the importance of each predictor. In particular, Variable Importance (VI)^41^ measures the contribution of each predictor to error reduction, offering insight into their overall role in the prediction process. However, VI does not provide meaningful information about the specific relationship between a predictor’s values and the corresponding predictions. To enhance the interpretability of RF’s predictive mechanism, we adopt surrogate tree models^43^. These are simple decision trees that approximate the behavior of a complex “black-box” model like a RF. To replicate the predictive mechanism of RF, surrogate trees are fitted using a new dataset denoted by ${D}_{n}^{*}={\left\{{\widehat{y}}_{i},{x}_{i}\right\}}_{i=1}^{n}$, where ${\widehat{y}}_{i}$ is the prediction obtained by the RF algorithm for the *i*-th observation. Surrogate trees are transparent and can be represented graphically, allowing for an examination of their structure (e.g., the splits), and the distribution of training observations across their terminal nodes. The main hyperparameter of surrogate trees is represented by the depth, which can be regarded as a measure of complexity as it is related to the number of terminal nodes (e.g., a depth of 1 corresponds to a tree with 2 terminal nodes). Trees with a smaller depth are more straightforward to be interpreted; however, this can reduce the accuracy of the surrogate as it can be constrained to group heterogeneous observations into a small set of terminal nodes. Conversely, a deeper, more complex surrogate tree can better replicate the RF’s predictions, but at the cost of reduced interpretability.

### Intensity prediction equations and ground motion to intensity conversion equations

To validate the effectiveness of the RF method and demonstrate its advantages, it is essential to compare its performance against the established and widely used standards in the field.

One of the standard approaches for estimating macroseismic intensities is through the application of empirical equations, commonly referred to as IPEs. IPEs describe the relationship between seismic variables, distance and macroseismic intensity at a given location. Considering that intensity decreases with increasing distance from the earthquake’s hypocenter, IPEs are also known as attenuation laws. It is worth noting that IPEs allow for the estimation of seismic intensity using a limited number of variables; therefore, it is crucial that the employed variables are derived from high-quality, region-specific data. Regional specificity ensures that the IPEs are accurately calibrated for particular areas, as seismic characteristics can vary significantly due to geological differences across regions. Using IPEs that are not developed for the region of application may lead to biased estimates, highlighting the importance of employing appropriately tailored versions for reliable intensity predictions.

A further approach commonly used to estimate macroseismic intensity is by converting the maximum ground shaking values recorded by instruments using GMICEs. The ground motion variables commonly used in GMICEs are PGA and/or PGV. In contrast, PGD, another ground motion variable, is less frequently utilized, as it primarily relates to permanent ground deformation rather than the transient shaking that GMICEs seek to estimate.

In this work we compare the Machine Learning predicted intensities, with those provided by the IPE and GMICE models developed for the Italian territory and listed in Table 1.

**Table 1:** Intensity IPE and GMICE models, where *I* is the MCS expected intensity, *h* is the earthquake depth and *R*_*epi*_ is the epicentral distance, both measured in *km.*

Model class—denomination	Definition	References
IPE—Pasolini	$I={I}_{epi}-0.0086\cdot (D-h)-1.037\cdot (ln(D)-ln(h))$ where $D=\sqrt{{{R}_{epi}}^{2}+{(3.91)}^{2}}$ ${I}_{epi}= -5.862+2.460\cdot {M}_{w}$	^44^
IPE—Sbarra	$I= -3.08\cdot log(D)+0.94\cdot {M}_{w}+4.47$ where $D=\sqrt{{{R}_{epi}}^{2}+{h}^{2}}$	^45^
IPE—Gomez	$I= 1.81-2.61\cdot log(D)-0.0039\cdot D+1.42\cdot {M}_{w}$ where $D=\sqrt{{{R}_{epi}}^{2}+{(9.87)}^{2}}$	^4^
GMICE—Faccioli	$I=8.69+1.80\cdot (log(PGV)-2)$	^46^
GMICE—Gomez	$I=4.514\cdot {e}^{0.502\cdot log(PGV)}$	^47^
GMICE—Faenza	$I=1.68+2.58\cdot log(PGA)\forall I\le 6$ $I=5.11+2.35\cdot log(PGV)\forall I>6$	^48^
GMICE—Oliveti_A	$I=3.01+0.86\cdot {log}^{2}(PGA)$	^49^
GMICE—Oliveti_V	$I=4.31+1.99\cdot log(PGV)+0.58\cdot {log}^{2}(PGV)$	^49^

### Model configurations and hyperparameter settings

The dataset of *n* = 5466 observations was randomly partitioned into a 70% training set (3827 observations) and a 30% test set (1639 observations). The RF model was estimated using the *randomForest* function from the R package of the same name^50^. We determined the optimal number of trees by monitoring the validation error on a set comprising 10% of the training data. The optimal number of trees thus obtained is 205. The number of predictors considered at each split was set to 2, following the standard rule where $m\approx \sqrt{P}$ ^51^ and $P$ = 6 is the total number of predictors. To address the class imbalance observed in the intensity data (as illustrated in Fig. 1), the *classwt* attribute of the *randomForest* function was employed to assign equal a priori weight to each intensity category. For the surrogate tree models, we used the *TreeSurrogate* function from the R package *iml*^52^. We configured three different depths (2,3 and 4), resulting in three different surrogate models referred to as S2, S3 and S4. This allows us to examine how varying levels of model complexity may influence predictive performance, interpretability, and uncertainty. Finally, the IPEs and GMICEs (Table 1) were implemented for comparison. To ensure a direct comparison with the RF and surrogate tree outputs, the resulting intensities for the test observations were rounded to the nearest integer.

### Model evaluation metrics

To evaluate the predictive performance of the adopted methods, we used several standard metrics^51^, with equations for a binary classification problem given by:

2 $$\begin{array}{c}Accuracy= \frac{TP+TN}{TP+TN+FP+FN} \end{array}$$

3 $$\begin{array}{c}Sensitivity= \frac{TP}{TP+FN}\end{array}$$

4 $$\begin{array}{c}Precision=\frac{TP}{TP+FP}\end{array}$$

5 $$\begin{array}{c}Specificity=\frac{TN}{TN+FP}\end{array}$$

6 $$\begin{array}{c}F1 score=\frac{Sensitivity\cdot Precision}{Sensitivity+Precision}\end{array}$$

where TP (True Positive) and TN (True Negative) are the number of correctly classified positive and negative observations, while FP (False Positive) and FN (False Negative) are the number of incorrectly classified positive and negative observations, respectively. While *accuracy* (Eq. 2) is the most common index, defined as the ratio of correctly predicted observations to the total number of predictions, it can be misleading in cases of class imbalance. Therefore, additional metrics should be considered for a comprehensive evaluation. When the focus is on the positive class, *sensitivity* and *precision* are commonly used. *Sensitivity* (Eq. 3) measures the proportion of actual positive observations that are correctly identified, while *precision* (Eq. 4) indicates the proportion of positive predictions that are truly correct. With respect to the negative class, *specificity* (Eq. 5) measures the correct prediction rate for the negative class. Further indices can be derived by combining these metrics, as in the case of the *F1 score* (Eq. 6), provides a single, harmonized metric for the positive class by combining sensitivity and precision, those that usually are considered more important. All these indices yield values between zero and one, where a higher value indicates better performance.

In our case, given that $K = 8$ intensity classes, we converted our multi-class problem into a binary classification by grouping the observed intensities into two categories: “High” (intensities 5–8) and “Low” (intensities 1–4). This choice is justified by the difference between the diagnostic effects between these two groups, i.e. completely transient effects for lower intensities versus the potential for permanent effects for higher intensities, with the probability of such effects increasing from intensity 5 onwards. For our evaluations, the “High” category is designated as the positive class.

### Indexes for evaluating prediction uncertainty

Quantifying prediction uncertainty is crucial for building confidence in a model’s output and enabling its cautious application. As most Machine Learning models, including our surrogate trees, do not inherently provide uncertainty measures for their predictions, we propose using impurity indexes to assess this. The rationale is straightforward: a higher level of impurity within a terminal node indicates greater uncertainty for any prediction associated with that node. We adopted two well-established impurity measures for this purpose: the *classification error* rate (${E}_{j}$) and the *Gini index* (${G}_{j}$)^51^. The metrics are defined as follows, where $j$ refers to the generic terminal node ${R}_{j}$:

$${E}_{j}=1-\left({\widehat{p}}_{jk}\right)$$

$${G}_{j}={\sum }_{k=1}^{K}{\widehat{p}}_{jk}\left(1-{\widehat{p}}_{jk}\right) .$$

In particular, *K* is the number of categories of the response variable and ${\widehat{p}}_{jk}$ denotes the proportion of training observations with category *k* in the *j*-th terminal node. Both the indexes return values between zero and one, with smaller values indicating a lower level of uncertainty in the predictions.

## Results and discussion

### Comparative analysis of the obtained estimates

For each predictive model, an 8 × 8 (given *K* = 8) confusion matrix has been constructed to compare the observed test intensities with the predicted ones. The confusion matrix presented in Table 2, which pertains to RF, indicates that the observed low-intensity classes are usually correctly predicted, with the exception of 16 cases belonging to intensity 4 for which the prediction is 5 (see the red area). Conversely, for high intensities, the number of observations wrongly predicted as “Low” is 23 (19 intensity 5 are predicted as 4, 1 intensity 5 is predicted as intensity 3, and 3 intensity 6 are predicted as intensity 4, see the yellow area): this result for high intensities may be influenced by their limited presence in the training data. Overall, the test classification error is quite low (0.024).

**Table 2:**
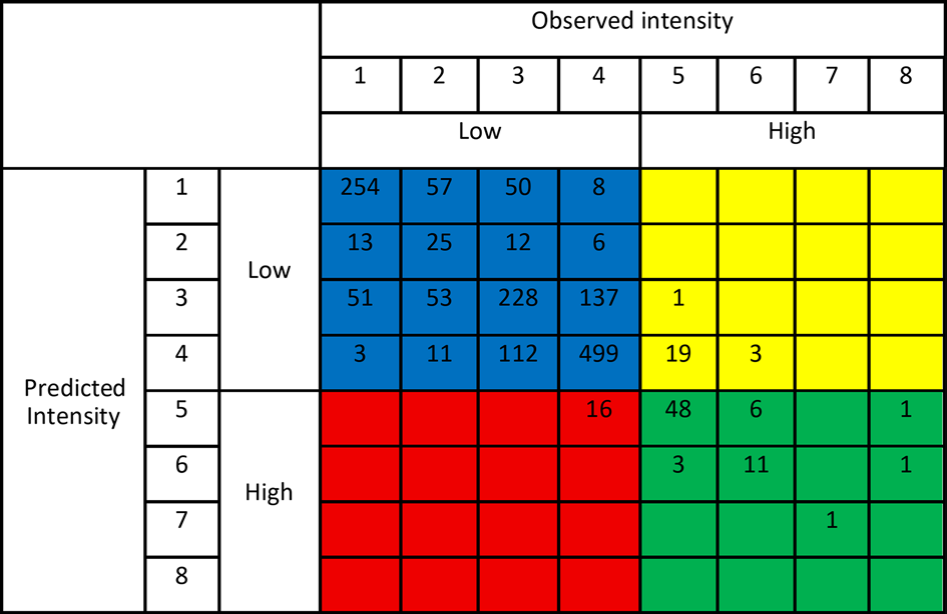
Confusion matrix for the test intensities obtained from RF. On the columns the observed intensity classes are reported, while on the rows the predicted ones. Colors indicate if the observation corresponds to TN (blue), FN (yellow), TP (green), and FP (red) when the classification problem is reduced to a binary classification problem with “Low” and “High” categories.

The test predictive performance metrics, based on the “High” vs. “Low” binary categorization of intensity is reported in Table 3 for all implemented methods: RF, surrogate trees, and the IPE and GMICE models named by the first author (see Materials and methods section). Bold characters identify the two highest values for each index. It is important to note that while multiple surrogate trees were estimated (i.e., with depth equal to 2, 3, and 4), they all exhibited identical predictive performance, so only a single entry is reported in Table 3 for this family of models. The RF model achieved the highest overall accuracy (0.976), followed by the surrogate trees, which exhibited only a 0.5% reduction in accuracy. Comparable accuracy levels were achieved by IPE-Pasolini and GMICE-Oliveti_V. The results for other performance measures indicate that IPEs and GMICEs generally have higher sensitivity than RF and the surrogate trees. However, their ability to correctly predict “High” intensities is weaker due to their comparatively low precision. An analysis of the F1 scores (a metric that prioritizes positive prediction performance, see Model evaluation metrics section), shows the RF with the highest value (0.785), followed by IPE–Pasolini and GMICE–Oliveti_V. This suggests that while Machine Learning models may predict fewer “High” intensities than other methods, their “High” predictions are more likely to correctly correspond to observed intensities greater than 4.

**Table 3:** The test predictive performance indexes for the considered predictive models in the case of “Low” vs “High” binary categories. With the GMICE-Oliveti_A and GMICE-Oliveti_V indices calculated respectively on a subset of 1411 and 1322 of the 1639 test observations, due to the presence of PGA or PGV values below the applicability limits of the functions.

Model evaluation metrics					
Method	Accuracy	Sensitivity	Precision	Specificity	F1
RF	**0.976**	0.755	**0.816**	**0.990**	**0.785**
Surrogate tree	**0.971**	0.500	**0.979**	**0.999**	0.662
IPE-Pasolini	0.962	0.830	0.629	0.970	**0.716**
IPE-Sbarra	0.942	0.872	0.497	0.946	0.633
IPE-Gomez	0.929	0.904	0.440	0.930	0.592
GMICE-Faccioli	0.874	**0.957**	0.308	0.869	0.466
GMICE-Gomez	0.942	0.904	0.497	0.944	0.642
GMICE-Faenza	0.909	**0.947**	0.382	0.907	0.544
GMICE-Oliveti_A	0.936	0.862	0.513	0.942	0.643
GMICE-Oliveti_V	0.948	0.872	0.590	0.954	0.704

Bold characters identify the two highest values for each index.

A joint assessment of the five indices (Table 3) reveals five distinct performance profiles: (1) overall-best (RF)**:** highest accuracy and F1 score, with high specificity and precision; (2) conservative high-precision (Surrogate)**:** high accuracy and exceptional precision/specificity, at the cost of lower sensitivity; (3) balanced (IPE-Pasolini; GMICE-Oliveti_V)**:** competitive accuracy and F1, with moderate precision/specificity; (4) Sensitivity-forward (e.g., IPE-Sbarra; IPE-Gomez): higher sensitivity with modest precision, yielding average F1; (5) Sensitivity-max (e.g., GMICE-Faenza; GMICE-Faccioli): very high sensitivity but low precision, resulting in the lower overall discriminative quality. Based on these profiles, RF is preferred for prompt and reliable mapping, while the surrogate tree is indicated for situations where minimizing false alarms is critical. The IPE-Pasolini and GMICE-Oliveti_V remain the most reliable classical references, while the remaining sensitivity-oriented models are appropriate when false positives are acceptable.

**Fig. 2:**
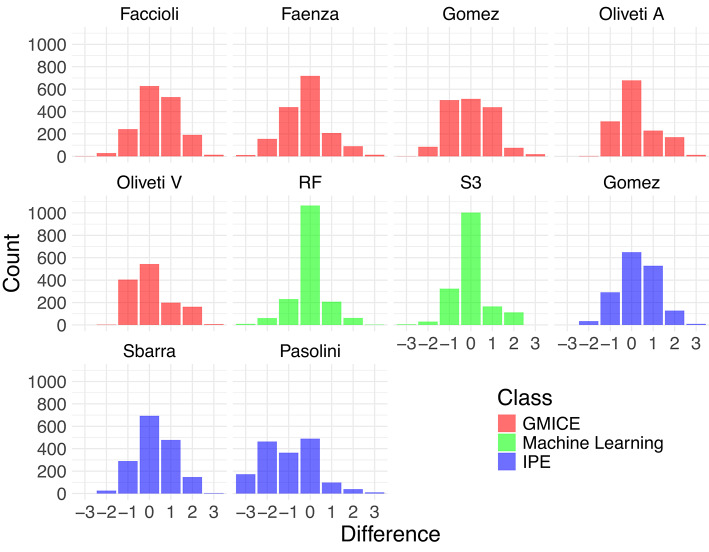
Bar plots showing the distribution of the intensity difference (for the test observations) between the predicted and the observed intensity category for all the employed models reported in Table 3. Note that the surrogate tree predictions are given by S3. The bar plots are color-coded to represent the model class: red for GMICE, green for Machine Learning and blue for IPE.

Beyond classification accuracy, i.e., determining whether the predicted class corresponds to the observed class, we also considered the distribution of the difference between predicted and observed intensities. This value is indicative of the number of classes that differentiate the predicted from the observed intensity, with a positive value, denoting that the predicted class is higher than the observed class, and a negative value indicating the opposite. As shown in Fig. 2, for all models, the most frequent outcome is a zero modal difference, indicating that predicted and observed intensities most commonly coincide. The GMICE set of models generally shows moderately peaked distributions centered around zero, indicating they are the least accurate of the methods. Within this group, Faenza and Oliveti_V show slightly narrower distributions. The Machine Learning models (RF and S3) exhibit a sharp peak at zero, demonstrating their high precision, with the surrogate tree showing a slightly wider spread. The IPE methods have broader distributions compared to Machine Learning methods but still maintain peaks near zero, with the exception of the Pasolini model.

Finally, we performed an auxiliary analysis investigating the use of M_wg_^38^ scale to possibly overcome the limitations of *M*_*w*_ in dealing with small-to-moderate magnitude events and we tested whether it would produce any significant change in our results (see details in Supplementary Information 1). We obtained no statistically significant differences in terms of model evaluation metrics for the adopted Machine Learning models. These results were expected by the nature of the transformation that has been implemented to move from *M*_*w*_ to M_wg_, given that RF is robust to linear transformation of the predictors^41^.

### Interpretability of machine learning models

The VI analysis (see the Materials and methods section) from the RF model (Fig. SI-1) indicates that ground motion measures (specifically GeoMean_pga and GeoMean_pgv) are more important for predicting seismic intensity than the source and distance parameters (focal_depth, Mw_trasf and logst_IPODIST). This finding is significant as the latter variables, used in traditional IPEs, are known to have limitations. They fail to capture the variability of ground motion caused by local site geology, source characteristics, and the direction of wave propagation. Consequently, traditional IPEs alone can lead to inaccurate intensity estimates and, when used in seismic hazard estimation procedures, can lead to incorrect results. To overcome these limitations, it is essential to consider ground motion measures more directly related to the intensity of effects at a given location.

**Fig. 3:**
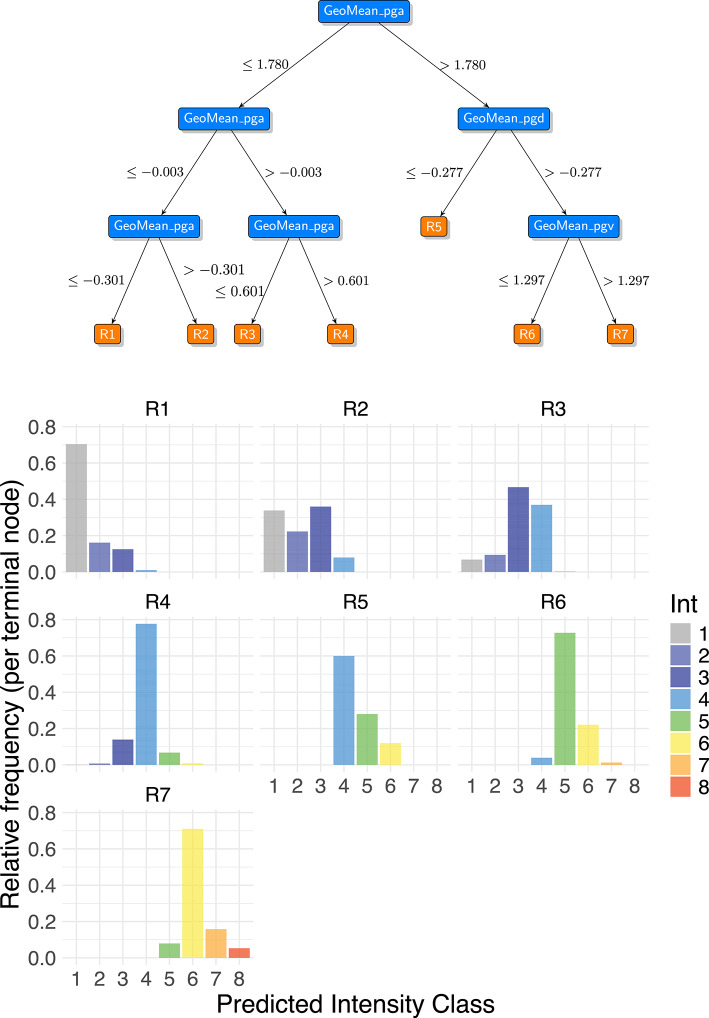
Top: surrogate tree of depth 3 (S3), wherein the rectangles represent the predictor for the split, and the conditions are displayed along the branches. Bottom: distribution of the intensity classes in the seven terminal nodes of S3 (${R}_{1}$, …, ${R}_{7}$).

The importance of ground motion measures is further confirmed by surrogate trees. As shown in the top panel of Fig. 3 the structure of the surrogate tree of depth 3 (S3) uses the most important RF predictors: GeoMean_pga, GeoMean_pgd and GeoMean_pgv. The tree also reveals complex interactions, such as multiple splits based on PGA values (left branch) and the use of all ground motion measures in a single branch (right branch). The S3 tree ends with 7 terminal nodes (${R}_{1}$- ${R}_{7}$) and the bottom panel of Fig. 3 illustrates the distribution of intensity classes within them. The first four terminal nodes (${R}_{1}$, ${R}_{2}$, ${R}_{3}$, ${R}_{4}$) are dominated by low intensity observations, while the remaining three (${R}_{5}$, ${R}_{6}$, ${R}_{7}$) are characterized by higher intensities. Specifically, intensity classes 1, 5, and 6 are the modal category in a single node each (${R}_{1}$, ${R}_{6}$, and ${R}_{7}$, respectively), while intensities 3 and 4 are the mode in two separate nodes (${R}_{2}$, ${R}_{3}$ and ${R}_{4}$, ${R}_{5}$, respectively). It is noteworthy that the other intensities (2, 7, and 8) never appear as node representatives, likely due to their infrequent occurrence in the dataset. As discussed in the Data selection and processing section, an imbalanced intensity distribution may negatively impact the model’s predictive capacity, particularly leading to the underestimation of higher intensities. This is evident in the lower sensitivity than specificity observed for both RF and its surrogates. Notably, in this application, it is never possible to generate predictions covering all eight possible intensity categories, even when RF is used or increased surrogate depth. The structure and intensity distributions observed in the terminal nodes of S2 and S4 (Figs. SI-2 and SI-3) are similar.

It is interesting to note that the lowest intensities are identified by the variable GeoMean_pga, while the high ones (terminal nodes ${R}_{5}$, ${R}_{6}$, ${R}_{7}$) require the intervention of GeoMean_pgv, in agreement with other authors^48, 53,54^, and GeoMean_pgd. The PGA is in fact a measure of shaking at high frequencies, which usually determines the perception of an earthquake, while the PGV is related to the energy content of motion at medium–low frequencies, resulting in a stronger correlation with intensities characterized by damage to medium-rise buildings^55^. The PGD should perform similarly, as it is associated with permanent deformation and long period motion. Furthermore, the split value -0.3 for the logarithm of the PGA, which defines the upper bound of the terminal node ${R}_{1}$, aligns with the felt/not felt transition range proposed by Worden et al.^8^.

We found that surrogate trees demonstrate high overall accuracy with respect to the prediction of the observed intensities (0.971, second-best in Table 3), while their capability in reproducing the RF predictions is considerably lower (an accuracy between 0.560 and 0.610 on training data and between 0.628 and 0.743 on test observations, depending on the surrogate’s depth).

Table 4 provides the predicted category (${\widehat{y}}^{*}$, given by the modal class) and the two impurity measures (E and G, introduced in the Materials and methods section), for each terminal node of the surrogate trees. These indices/measures, which range from 0 to 1, serve as a measure of prediction of uncertainty, with values exceeding 0.5 suggesting lower reliability. An analysis of the uncertainty values in Table 4 reveals that the uncertainty measures vary in relation to the complexity of the paths, in turn influenced by the depth of the surrogate tree. Deeper trees generally lead to more confident predictions. For instance, the average E and G values for S4 (0.386 and 0.499, respectively) are lower than those for S2 (0.442 and 0.580) and S3 (0.393 and 0.514). This suggests that the more complex a surrogate tree becomes, the better it can classify training observations, leading to lower average uncertainty, even if some differences among the nodes of the same surrogate tree are observed. However, it is essential to consider the trade-off between these improvements and the probability of overfitting the training data, as this can adversely impact the predictions made on test observations.

**Table 4:** Summary information for the terminal nodes of surrogate trees (S2, S3 and S4): intensity prediction${\widehat{y}}^{*}$, classification error rate (E) and Gini index (G).

Surrogate Tree	Terminal node	${\widehat{y}}^{*}$	E	G
S2	${R}_{1}$	1	0.445	0.607
	${R}_{2}$	4	0.436	0.580
	${R}_{3}$	4	0.400	0.547
	${R}_{4}$	5	0.487	0.586
S3	${R}_{1}$	1	0.296	0.463
	${R}_{2}$	3	0.640	0.700
	${R}_{3}$	3	0.533	0.632
	${R}_{4}$	4	0.223	0.373
	${R}_{5}$	4	0.400	0.547
	${R}_{6}$	5	0.273	0.421
	${R}_{7}$	6	0.289	0.461
S4	${R}_{1}$	1	0.229	0.380
	${R}_{2}$	1	0.462	0.608
	${R}_{3}$	3	0.640	0.700
	${R}_{4}$	3	0.518	0.669
	${R}_{5}$	4	0.516	0.559
	${R}_{6}$	4	0.194	0.326
	${R}_{7}$	4	0.453	0.532
	${R}_{8}$	4	0.400	0.547
	${R}_{9}$	6	0.483	0.583
	${R}_{10}$	5	0.062	0.119
	${R}_{11}$	6	0.289	0.461

### Out-of-sample single-earthquake case study

**Fig. 4:**
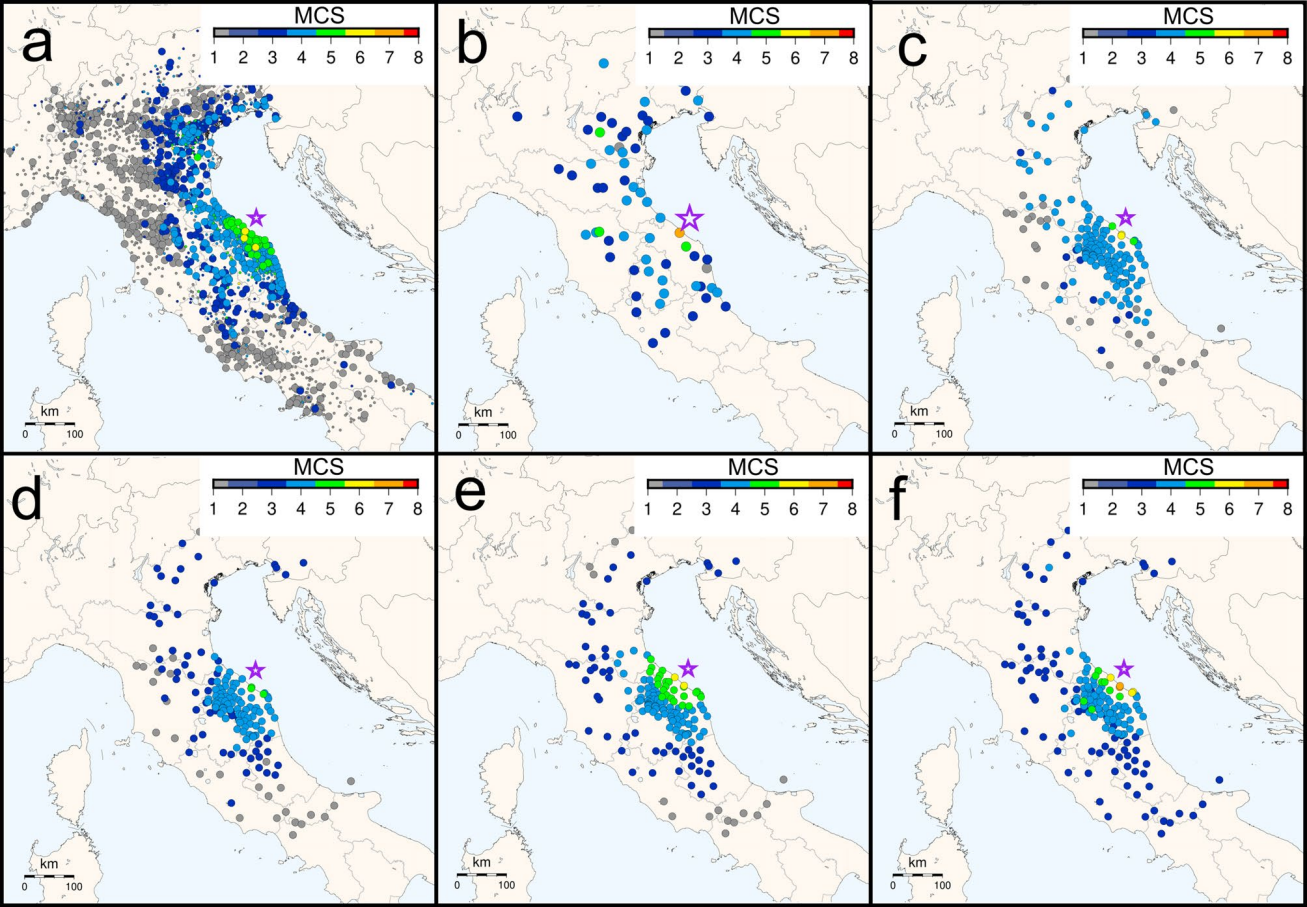
Maps of the intensity for the Pesaro-Urbino Coast earthquake (November, 9th 2022, 5.5 *M*_*w*_) with purple star representing the epicenter. (**a**) HSIT municipality intensities assessed with all the 12,354 reports, small dots refer to values assessed with less than 3 reports. (**b**) HSIT values assessed using only the questionnaires received during the first 15 min after the earthquake. Estimated intensity values for every seismic station obtained by applying respectively: (**c**) RF, (**d**) surrogate S3, (**e**) IPE-Gomez, (**f**) GMICE Oliveti_V.

We applied our framework to a real-world scenario using the November 9, 2022, Adriatic Sea earthquake (*M*_*w*_ 5.5) located near Pesaro-Urbino. This event was the strongest in Italy in 2021–2022 and was widely felt by citizens, generating over 12,000 public reports in the following days with which the MCS intensities of more than 2200 municipalities were estimated. The resulting intensity map (Fig. 4a) was compiled from the HSIT system (https://e.hsit.it/33301831/index.html)^24,56^ using all available reports, 94% of which collected within the first 24 h. The availability of a significant amount of HSIT data further supports the selection of this seismic event.

The predictive process begins within minutes of an earthquake’s detection, once the seismologists have estimated its magnitude and location. In our case, the necessary predictors for the models were available approximately 15 min post-event. At this point, only about 90 questionnaires had been submitted to the HSIT system, resulting in a very sparse intensity map (Fig. 4b). Our predictive models RF and S3 (trained on all the available data described in section Materials and methods), along with IPE-Gomez and GMICE-Oliveti_V equations (see Table 1), were used to generate comprehensive intensity maps. Once all the data are available, the time required to process the data, generate predictions, and produce the final maps was just 1.94 s on a standard computer (equipped with an AMD Ryzen 5 3600 6-Core Processor 3.60 GHz, no core parallelization, and 16 GB of RAM), demonstrating the framework’s suitability for real-time application.

The prediction maps derived from RF and S3 for the 5 km radius areas around monitoring stations are reported in Fig. 4c and d. These predictions show considerable similarity to the final intensity distributions from the HSIT system (Fig. 4a), which is characterized by an inherent latency being the results of some days of data collection. This proves the value of our models, as they can provide a detailed overview of the earthquake’s impact within minutes of its occurrence, in contrast to the sparse information available in the first 15 min (Fig. 4b). Both the RF and S3 maps successfully identify the overall earthquake perception area and the extent of intensity 4. This makes them crucial for their intended purpose: enabling emergency coordinators to make decisions in the first few minutes after a seismic event based on the expected intensity in a given area.

We also examined prediction maps from traditional IPE and GMICE models listed in Table 2. The two most recently published examples of the respective groups are shown, namely IPE-Gomez (Fig. 4e) and GMICE-Oliveti_V (Fig. 4f). The IPE models generally agree with observed intensities for this specific earthquake, but systematically underestimate the northern Adriatic coastal intensities and fail to predict intensity 1 at greater distances (south and west, Fig. 4e). Conversely, the majority of GMICE models tend to overestimate maximum intensities and to underestimate intensities north of the epicenter (Fig. 4f). For a quantitative evaluation of the predictions please refer to the bar plots of the differences between predicted intensities and observed reported in Fig. SI-4 and the model evaluation metrics contained in Table SI-1. Furthermore, Fig. SI-5, Fig. SI-6 and Table SI-2 show the predictions and their evaluation for the Accumoli earthquake occurred in 2016 (*M*_*w*_ 6.0), a seismic event that caused extensive damage.

## Conclusion

In this study, we introduce a predictive framework that leverages Machine Learning for the rapid and interpretable estimation of macroseismic intensity. Unlike traditional empirical models, our approach models complex, non-linear relationships and recognizes the inherently qualitative nature of the MCS scale. The predictive performance of our method was evaluated against several IPEs and GMICEs developed for Italy.

We found that the Random Forest model, paired with surrogate trees for explainability, not only achieved superior predictive performance compared to existing IPEs and GMICEs but also provided a transparent methodology for experts and decision-makers. Our key innovation lies in the integration of model explainability and uncertainty quantification into a single, cohesive framework. Specifically, variable importance and surrogate trees were used to elucidate the RF model’s predictive mechanism, and we incorporated uncertainty quantification using node impurity measures to assess the reliability of predictions.

Our results also highlight the utility and limitations of employing surrogate trees to explain RF predictions. Although the surrogate trees do not perfectly reproduce the RF predictions, they demonstrate high overall accuracy and also allow for a representation of the RF, although considerably simplified, which could be useful for visualizing the process even for non-experts. A notable limitation remains the lack of a standardized metric in the literature to assess a surrogate’s fidelity to the original black-box model, reflecting the current state of research rather than a flaw in our method. The analysis also revealed a data imbalance issue, as our models tended to underestimate high macroseismic intensities due to their natural infrequency.

Future work should focus on addressing this challenge to further enhance the models’ predictive power for high-impact earthquakes. We remark that the proposed predictive method is generalizable and can be adapted to include alternative variables (e.g., different intensity or magnitude scales, other ground motion measures) depending on the focus regions and available dataset.

Ultimately, this research provides a transparent and powerful tool that can be rapidly implemented for real-time seismic hazard applications, bridging the gap between advanced data science and critical decision-making in seismology.

## Supplementary Information

Below is the link to the electronic supplementary material.

Supplementary Material 1

Supplementary Material 2

## Data Availability

The datasets generated during and/or analyzed during the current study are available in the GitHub repository named Random-Forest-for-Seismic-Intensity-estimation, https://github.com/LucaPate/Random-Forest-for-Seismic-Intensity-estimation.

## Code availability

The code developed and utilized in this study were uploaded to the open-source GitHub repository. The code can be accessed via the following link: https://github.com/LucaPate/Random-Forest-for-Seismic-Intensity-estimation.
