# Correction: Machine learning for prompt estimation of macroseismic intensity from seismometric data in Italy

**DOI:** 10.1038/s41598-026-44475-8

**Published:** 2026-03-17

**Authors:** Luca Patelli, Michela Cameletti, Valerio De Rubeis, Nicola Alessandro Pino, Claudia Piromallo, Paola Sbarra, Patrizia Tosi

**Affiliations:** 1 Department of Economics, University of Bergamo, Via dei Caniana 2, 24127 Bergamo, Italy; 2 Istituto Nazionale di Geofisica e Vulcanologia (INGV), Via di Vigna Murata 605, 00143 Roma, Italy; 3 School of Science and Technology, Geology Section, University of Camerino, Via Gentile III Da Varano 7, 62032 Camerino, Italy

Correction to: *Scientific Reports* 10.1038/s41598-026-35740-x, published online 04 February 2026

The original version of this Article contained a repeated error in the dataset used. The error has been corrected in the sections Materials and methods, References and Acknowledgements.

The original Article has been corrected.

