# Supplementary material for: Machine learning for prompt estimation of macroseismic intensity from seismometric data in Italy
Source: Sci Rep. 2026 Feb 4;16:7265. doi: 10.1038/s41598-026-35740-x (PMC12923720; doi:10.1038/s41598-026-35740-x)
Supplement: Supplementary file 1 — Supplementary Material 1 [file 41598_2026_35740_MOESM1_ESM.docx]

**Supplementary information 1**

**Machine Learning for Prompt Estimation of Macroseismic Intensity from Seismometric Data in Italy**

Luca Patelli^1*^, Michela Cameletti^1^, Valerio De Rubeis^2^, Nicola Alessandro Pino^3^, Claudia Piromallo^2^, Paola Sbarra^2^, Patrizia Tosi^2*^

^1^Department of Economics, University of Bergamo, Via dei Caniana, 2, Bergamo, 24127, Italy. ^2^Istituto Nazionale di Geofisica e Vulcanologia (INGV), Via di Vigna Murata 605, Roma, 00143, Italy. ^3^School of Science and Technology - Geology Section, University of Camerino, Via Gentile III Da Varano 7, Camerino, 62032, Italy.

## **Multi-earthquake validation-test**

The Variable Importance (VI, see the Materials and methods section) from the Random Forest (RF) is displayed in Fig. SI-1. Our results indicate that the variables describing the earthquake and the distance to the source (namely focal_depth, Mw_trasf and logst_IPODIST) are less important for predicting seismic intensity compared to GeoMean_pga and GeoMean_pgv, which play a dominant role.


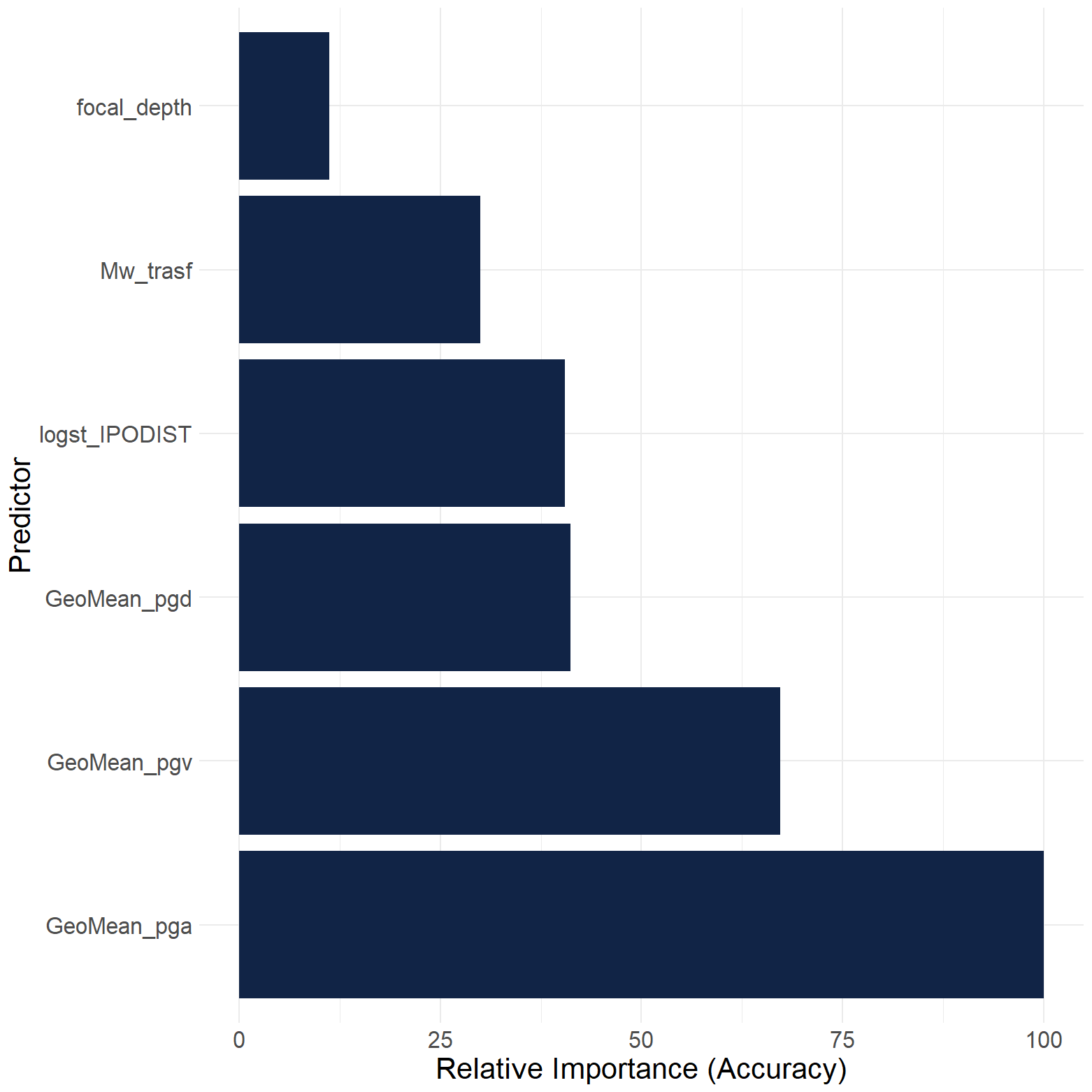


**Fig. SI-1.** Relative importance of the predictors from the RF (values are expressed relatively to the maximum).

In fact, the former variables used in traditional IPEs have limitations. They fail to capture the variability of ground motion due to factors such as detailed seismic source characteristics, geological properties of the site and the direction of seismic wave propagation. Consequently, the use of traditional IPEs alone can lead to under- or over-estimation of macroseismic intensity at a given site, with potential negative implications in terms of seismic hazard. To overcome these limitations, it is necessary to consider ground motion measures more directly related to the intensity of effects at a given location.

The surrogate of depth 2 is composed of 3 splits involving GeoMean_pga GeoMean_pgd, two of the most important predictors highlighted by the VI (see Fig. SI-1). The aforementioned splits result in a total of four terminal nodes, which, as illustrated in the bottom panel of Fig. SI-2, have modal classes comprising at least 50% of the training observations. A focus on the intensity classes characterizing each terminal node reveals a similarity in the intensities present at each leaf. The first terminal node is identified by the path GeoMean_pga *<*= 1.7804407 & GeoMean_pga *<*= -0.003006; the observations in this node are characterized by low seismic intensities, ranging from intensity 1 to 4, with a mode on class 1. The intensity distribution for the second terminal node ($R_{2}$) also includes classes 5 and 6, albeit to a marginal extent, equivalent to less than 5% of the observations. In contrast, nodes $R_{3}$ and $R_{4}$, which are defined by a split that considers GeoMean_pgd, exhibit a shift in the intensity distribution to higher levels, suggesting the expected onset of more disastrous effects.

Given that the dataset contains eight intensity classes, the presence of only four terminal nodes limits the predictions, preventing them from covering the entire range of intensity classes from 1 to 8. Consequently, an increase in the depth of the surrogate is necessary to try to obtain at least a terminal node for each intensity class.

An examination of the top panel of Fig. SI-3 reveals that S4 has a total of ten splits and terminates with eleven leaf nodes. From the root node to the leaf nodes, it can be observed that the splits involve from one to four different predictors. The terminal nodes displayed in the bottom panel illustrate that the left paths in the surrogate tree, which are solely based on GeoMean_pga, are characterized by intensity classes ranging from 1 to 4 (with the exception of $R_{4}$, where a small percentage of observations is represented by the intensity class 5). The inclusion of the predictor GeoMean_pgv in the paths leading to $R_{6}$ and $R_{7}$ results in a prevalence of high intensity classes within the intensity distributions. Starting from $R_{9}$ the modal intensity is higher than 4. With the exception of $R_{3}$, $R_{4}$ and $R_{5}$, the remaining nodes exhibit a clear modal class with a relative frequency above 0.50. Even in this more complex setting, it appears that not all the intensity classes are the predictions of at least one terminal node; this is the case of intensities 2, 7 and 8, which are intensity classes with specific reasons for under-reporting (as explained earlier).

**Fig. SI-2.** Top: surrogate tree of depth 2 (S2), wherein the rectangles represent the predictor for the split, and the conditions are displayed along the branches. Bottom: distribution of the intensity classes in the four terminal nodes of S2 ($R_{1}$, ..., $R_{4}$).




**Fig. SI-3.** Top: surrogate tree of depth 4 (S4), wherein the rectangles represent the predictor for the split, and the conditions are displayed along the branches. Bottom: distribution of the intensity classes in the eleven terminal nodes of S4 ($R_{1}$, ..., $R_{11}$).

## **Pesaro-Urbino earthquake out-of-sample case study**

From the Pesaro-Urbino case study we have 219 observations. For all of these observations the values of the six regressors available, while the intensity class is available only for 82 of them. This is due to the availability of filled HSIT questionnaires, as detailed in the main text (see the Data selection and processing section). As illustrated in Fig. 4, predictions were obtained for all the observations. However, the differences between the observed and predicted intensity class were computed only for the 82 complete observations, for which both the regressors and the response variable are available.


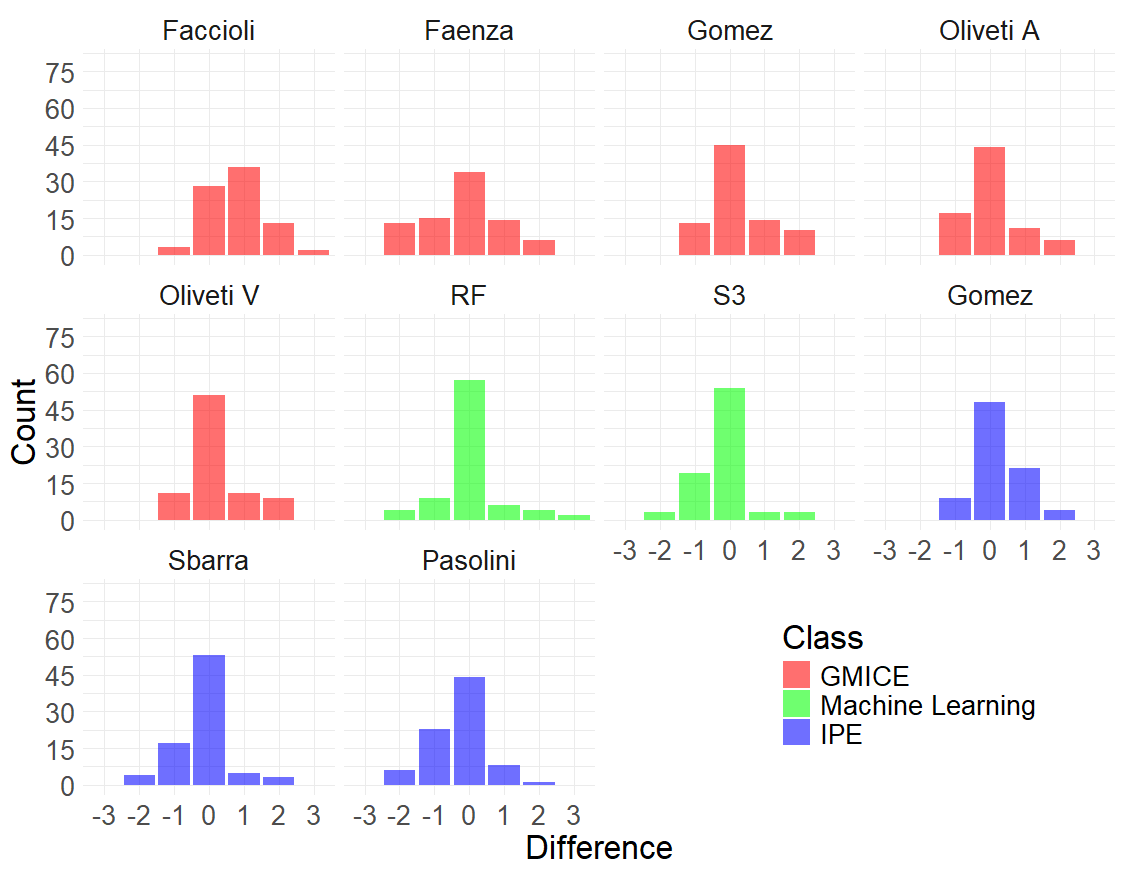


**Fig. SI-4.** Bar plots showing the distribution of the intensity difference (for the Pesaro-Urbino observations) between the predicted and the observed intensity category for all the employed models reported in Table 3. Note that the surrogate tree predictions are given by S3. The bar plots are color-coded to represent the model class: red for GMICE, green for Machine Learning and blue for IPE.

The results seem similar to those reported in Fig. 2. All the models tend to have a zero modal difference. In particular, RF and the surrogate of depth 3 appear to be the methods with the highest peaks at zero. These are followed by the IPE proposed by Sbarra. In terms of differences, RF reaches differences also of +3, while S3 shows a maximum positive difference of +2. However, the latter, compared to RF, presents a higher number of underpredictions corresponding to a difference equal to -1. GMICE models tend to have higher counts for positive differences (overpredictions), while IPEs show different behaviors: Gomez has higher counts for positive differences, while Sbarra and Pasolini in negative ones (underpredictions).

The predictive model evaluation metrics for RF and S3 are reported in Table SI-1. These values align with those presented in the main text of the paper (see Table 3), as RF exhibits greater accuracy and sensitivity than S3. Both the ML models have the same high precision and specificity, but RF appears to be a better classifier for high intensities than S3, as indicated by its higher F1 score.

| **Model evaluation metrics** | | | | | |
| --- | --- | --- | --- | --- | --- |
| **Method** | **Acc.** | **Sens.** | **Prec.** | **Spec.** | **F1** |
| RF | 0.976 | 0.755 | 1 | 1 | 0.800 |
| S3 | 0.963 | 0.500 | 1 | 1 | 0.667 |

Acc. = accuracy , Sens. = sensitivity, Prec. = precision, Spec. = specificity

**Table SI-1.** The test predictive performance indexes for RF and S3 models in the case of “Low” vs “High” binary categories. The indices are calculated on a subset of 82 observations out of the 219 available test observations.

## **Accumuli earthquake out-of-sample case study**

We applied our framework to a second real-world scenario using the August 24, 2016, Accumuli earthquake (Mw 6). To train our Machine Learning models (i.e., RF and the three surrogates) the data presented in the Data selection and processing section were used, but excluding now the 312 observations referring to the Accumuli earthquake. For all these observations the six regressors are available, but only 130 of them have the corresponding averaged intensity class. This is due to the availability of filled HSIT questionnaires, as detailed in the main text (see the Data selection and processing section). Intensity predictions were obtained for all the 312 observations (see Fig. SI-5) while differences between the observed and predicted intensity class were computed for the 130 complete observations.

**
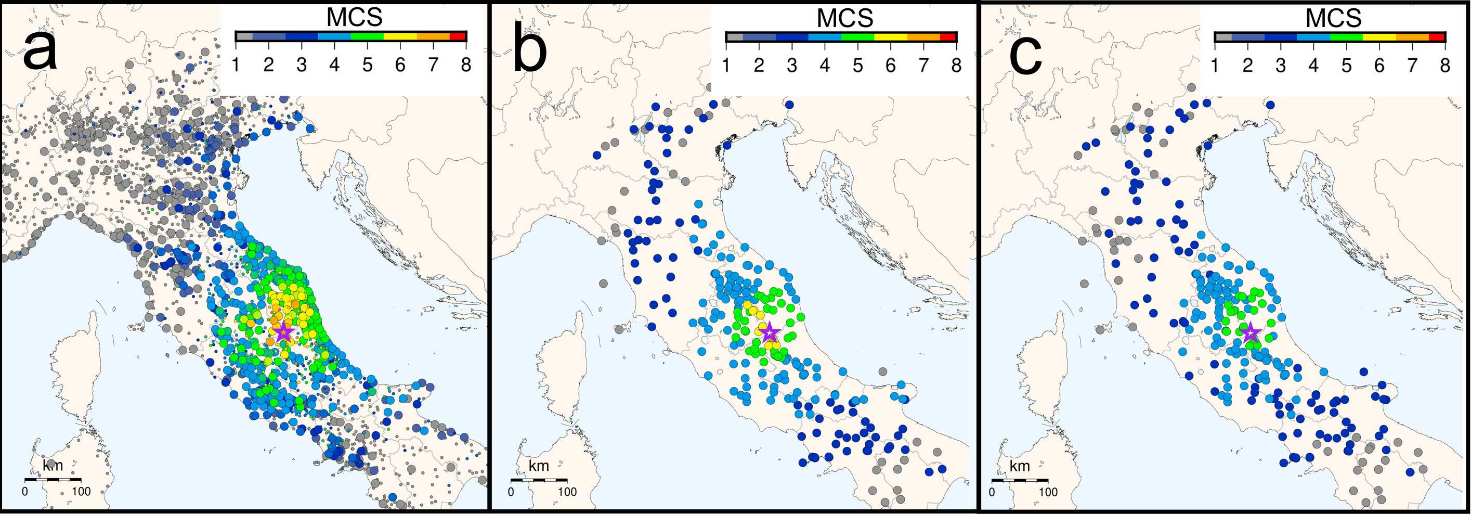
**

**Fig. SI-5.** Maps of the intensity for the Accumuli earthquake (August, 24th 2016, 6 *M_w_*) with purple star representing the epicenter. (a) HSIT municipality intensities assessed with all the 10681 reports, small dots refer to values assessed with less than 3 reports. Estimated intensity values for every seismic station obtained by applying respectively: (b) RF, (c) surrogate S3.


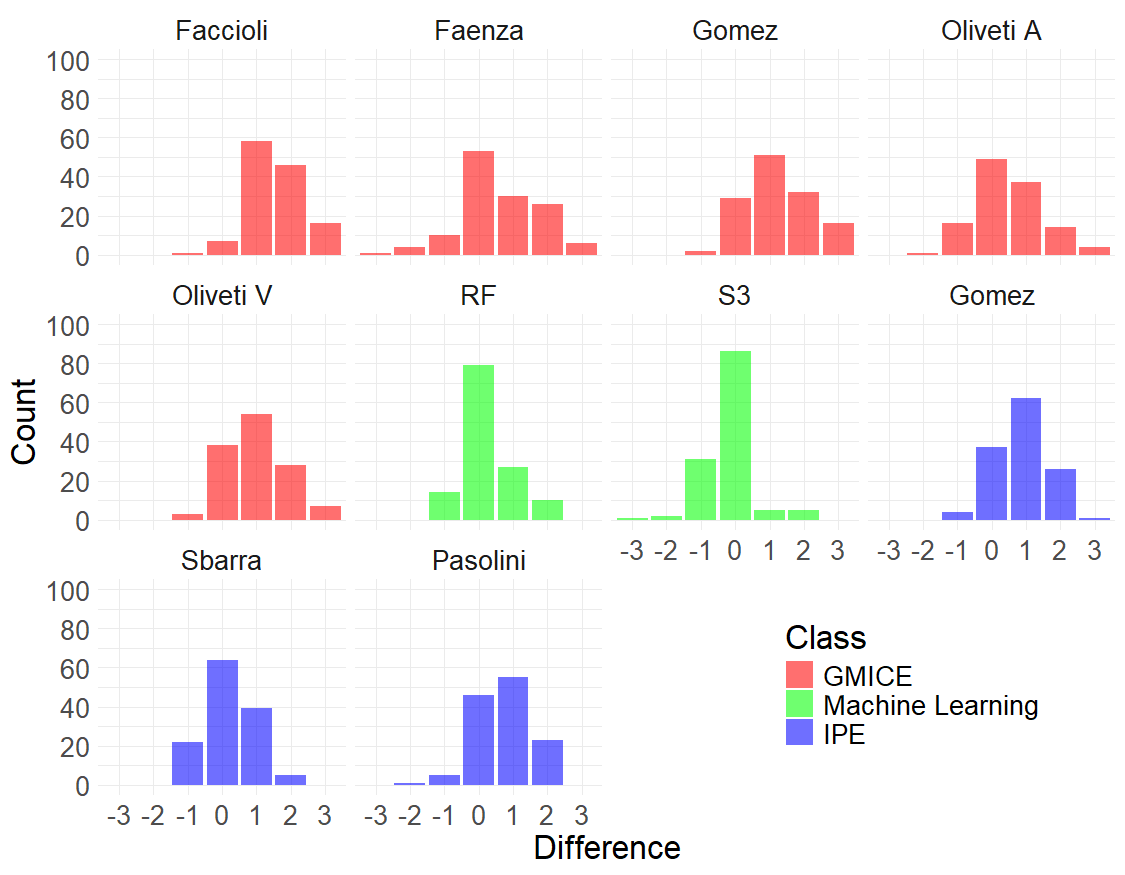


**Fig. SI-6.** Bar plots showing the distribution of the intensity difference (for the Accumuli observations) between the predicted and the observed intensity category for all the employed models reported in Table 3. Note that the surrogate tree predictions are given by S3. The bar plots are color-coded to represent the model class: red for GMICE, green for Machine Learning and blue for IPE.

The results for this earthquake are quite different with respect to the case studies reported previously. Only a limited number of models exhibit a zero-modal difference, including RF, S3, the GMICE of Faenza and Oliveti A, and the IPE of Sbarra. In particular, RF and S3 show the highest peaks at zero. The remaining models have a tendency to overpredict the intensities, with peaks on positive differences and few counts on the negative ones. In terms of the range of differences, RF has the narrowest range (with differences falling between -1 and +2), while S3 also has differences equal to -2 and -3. In particular, S3 is characterized by a higher frequency of underpredictions corresponding to -1, while for RF has a higher number of overpredictions equal to +1 is observed (compared to S3).

The predictive model evaluation metrics for RF and S3 are reported in Table SI-2. These values are similar to those presented in the main body of the paper (see Table 3), with RF demonstrating higher accuracy and sensitivity than S3. Both ML models have high precision and specificity, but S3 achieves higher levels. Looking at the F1 score, it can be concluded that, for high intensities, RF is preferable to S3.

| **Model evaluation metrics** | | | | | |
| --- | --- | --- | --- | --- | --- |
| **Method** | **Acc.** | **Sens.** | **Prec.** | **Spec.** | **F1** |
| RF | 0.877 | 0.792 | 0.864 | 0.927 | 0.826 |
| S3 | 0.815 | 0.512 | 0.962 | 0.988 | 0.676 |

Acc. = accuracy , Sens. = sensitivity, Prec. = precision, Spec. = specificity

**Table SI-2.** The test predictive performance indexes for RF and S3 models in the case of “Low” vs “High” binary categories. The indices are calculated on a subset of 130 observations out of the 312 available test observations.
