# Supplementary material for: Machine learning for prompt estimation of macroseismic intensity from seismometric data in Italy
Source: Sci Rep. 2026 Feb 4;16:7265. doi: 10.1038/s41598-026-35740-x (PMC12923720; doi:10.1038/s41598-026-35740-x)
Supplement: Supplementary file 2 — Supplementary Material 2 [file 41598_2026_35740_MOESM2_ESM.docx]

**Supplementary information 2**

**Machine Learning for Prompt Estimation of Macroseismic Intensity from Seismometric Data in Italy**

Luca Patelli^1*^, Michela Cameletti^1^, Valerio De Rubeis^2^, Nicola Alessandro Pino^3^, Claudia Piromallo^2^, Paola Sbarra^2^, Patrizia Tosi^2*^

^1^Department of Economics, University of Bergamo, Via dei Caniana, 2, Bergamo, 24127, Italy. ^2^Istituto Nazionale di Geofisica e Vulcanologia (INGV), Via di Vigna Murata 605, Roma, 00143, Italy. ^3^School of Science and Technology - Geology Section, University of Camerino, Via Gentile III Da Varano 7, Camerino, 62032, Italy.

## **Test for the use of Mwg scale**

To test the use of Mwg scale (more appropriate for representing radiated seismic energy in small to moderate events) we applied the transformation to $M_{w}$ obtain the Das Magnitude Scale (Mwg, Das et al., 2019). For practical implementation, the transformation is:

$Mwg=1.1 M_{w} - 0.88$.

We then performed the analysis and obtained the following results.

**
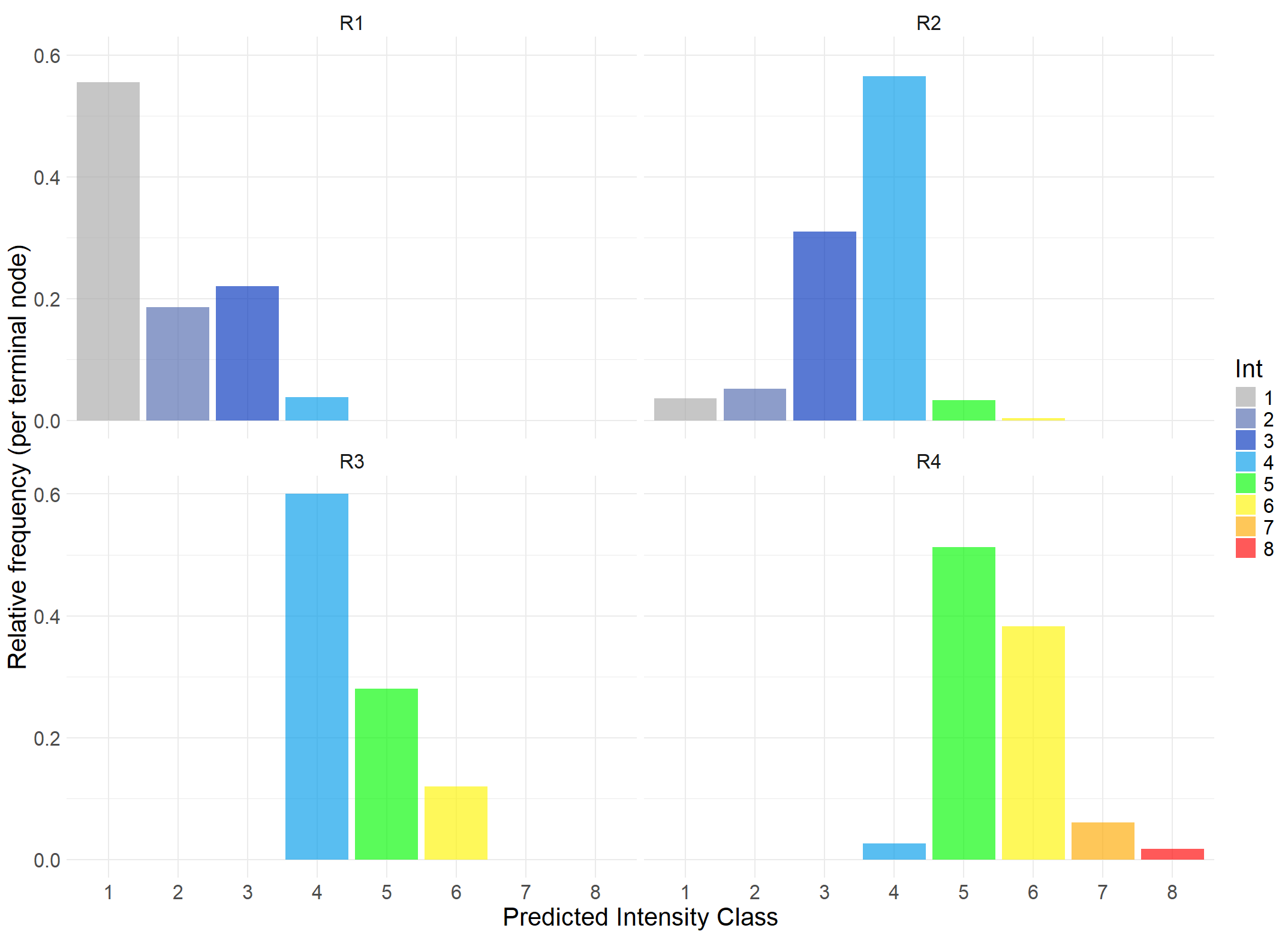

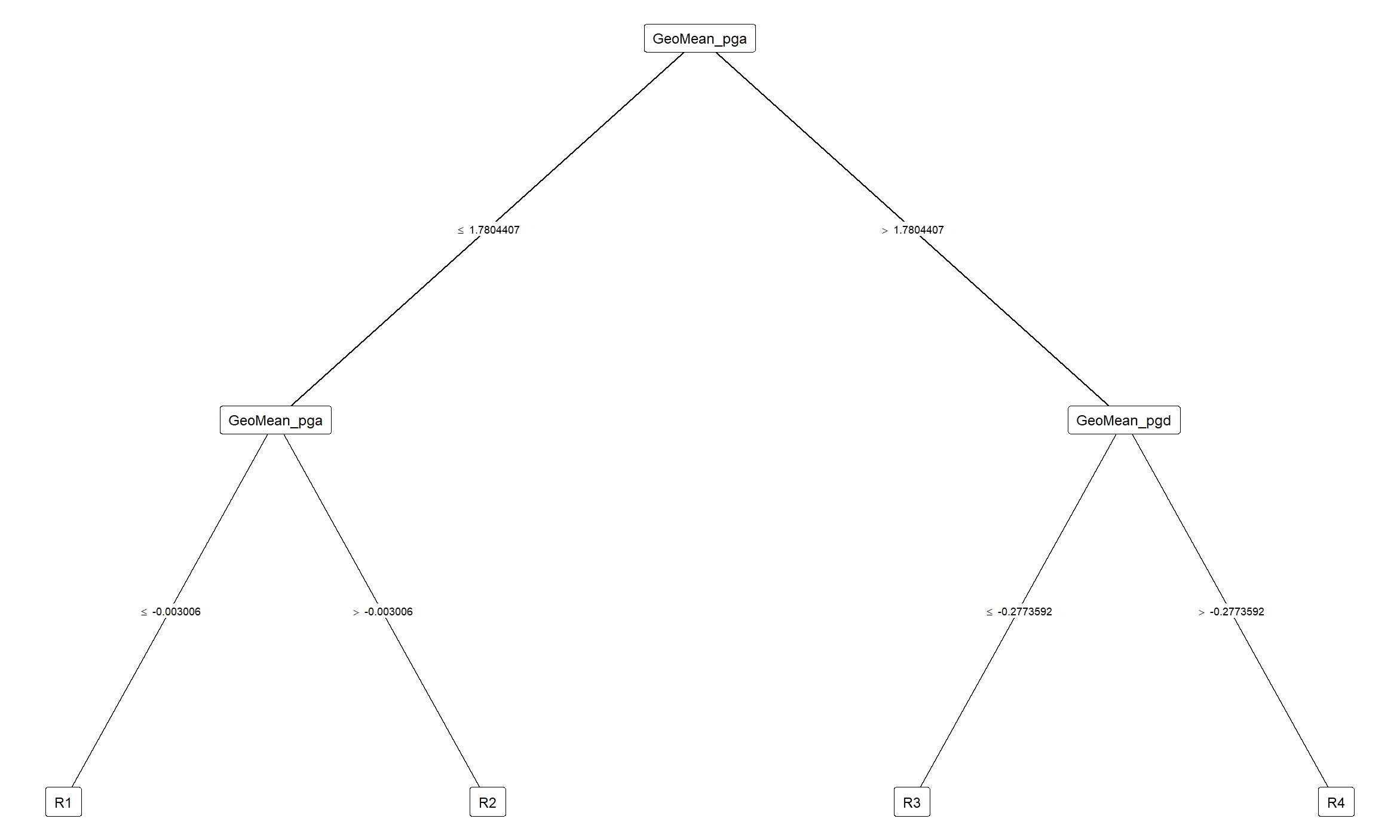
Fig. SI-2-1.** Top: surrogate tree of depth 2 (S2), wherein the rectangles represent the predictor for the split, and the conditions are displayed along the branches. Bottom: distribution of the intensity classes in the four terminal nodes of S2 ($R_{1}$, ..., $R_{4}$).

**
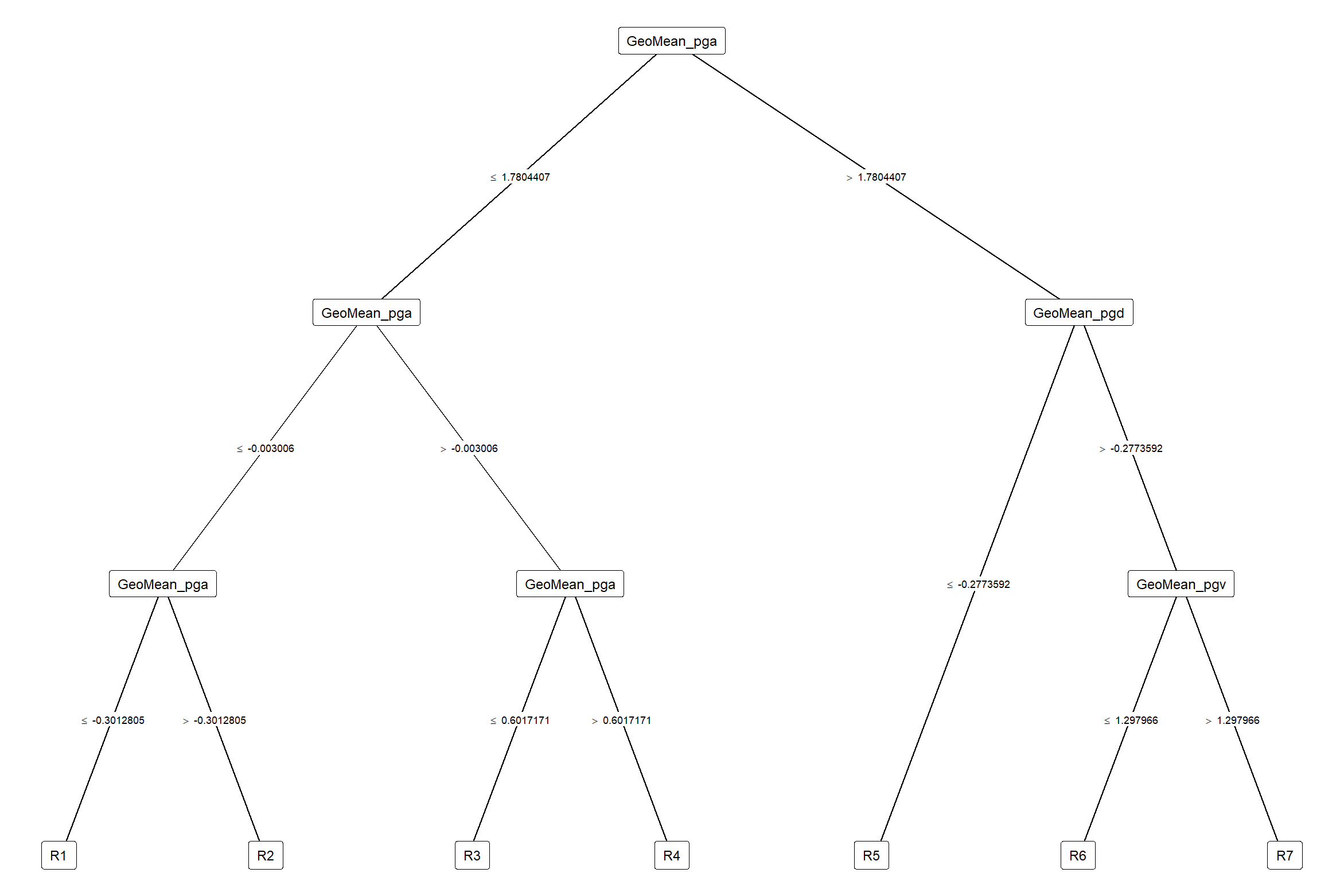

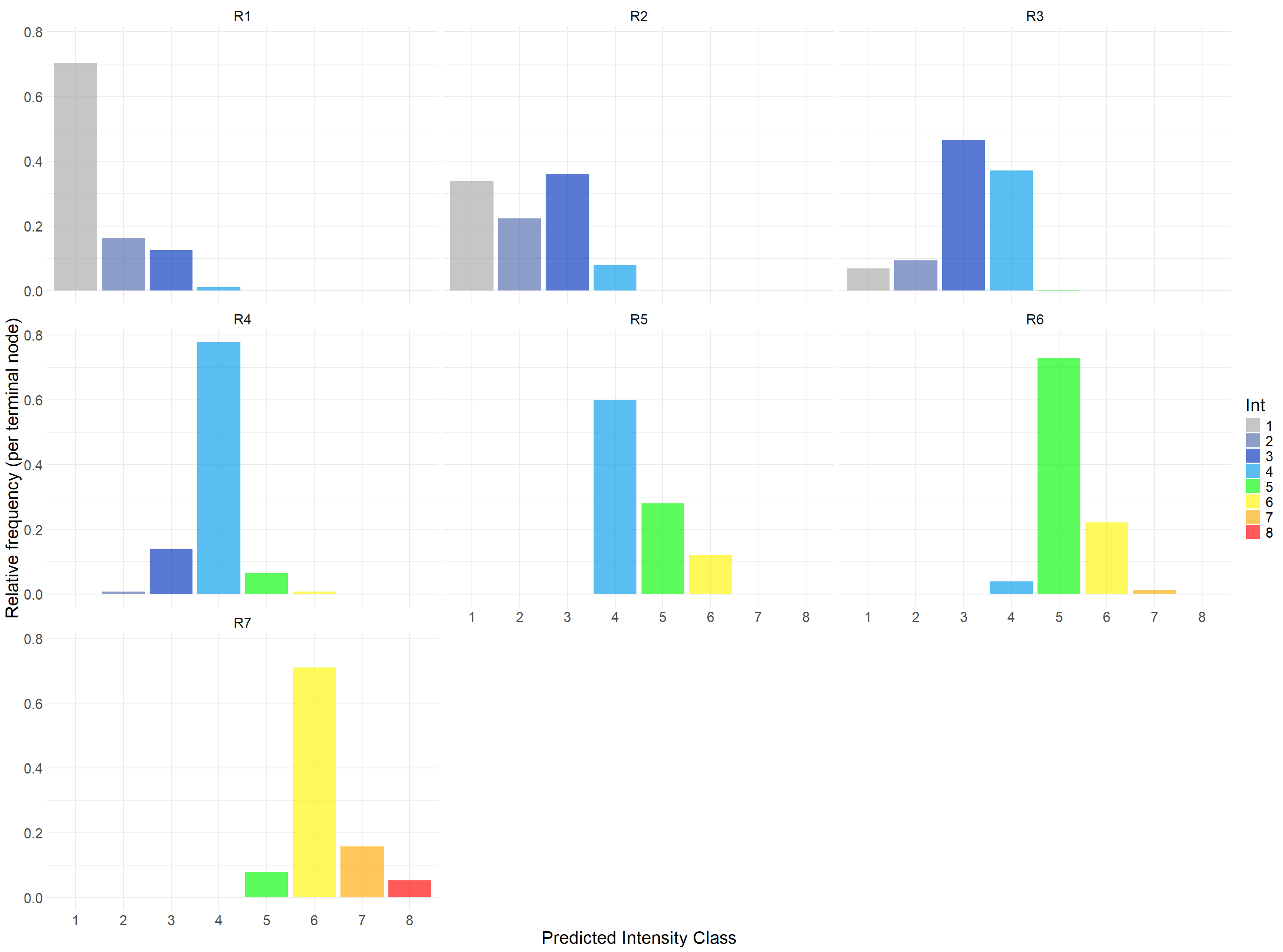
Fig. SI-2-2.** Top: surrogate tree of depth 3 (S3), wherein the rectangles represent the predictor for the split, and the conditions are displayed along the branches. Bottom: distribution of the intensity classes in the seven terminal nodes of S3 ($R_{1}$, ..., $R_{7}$).


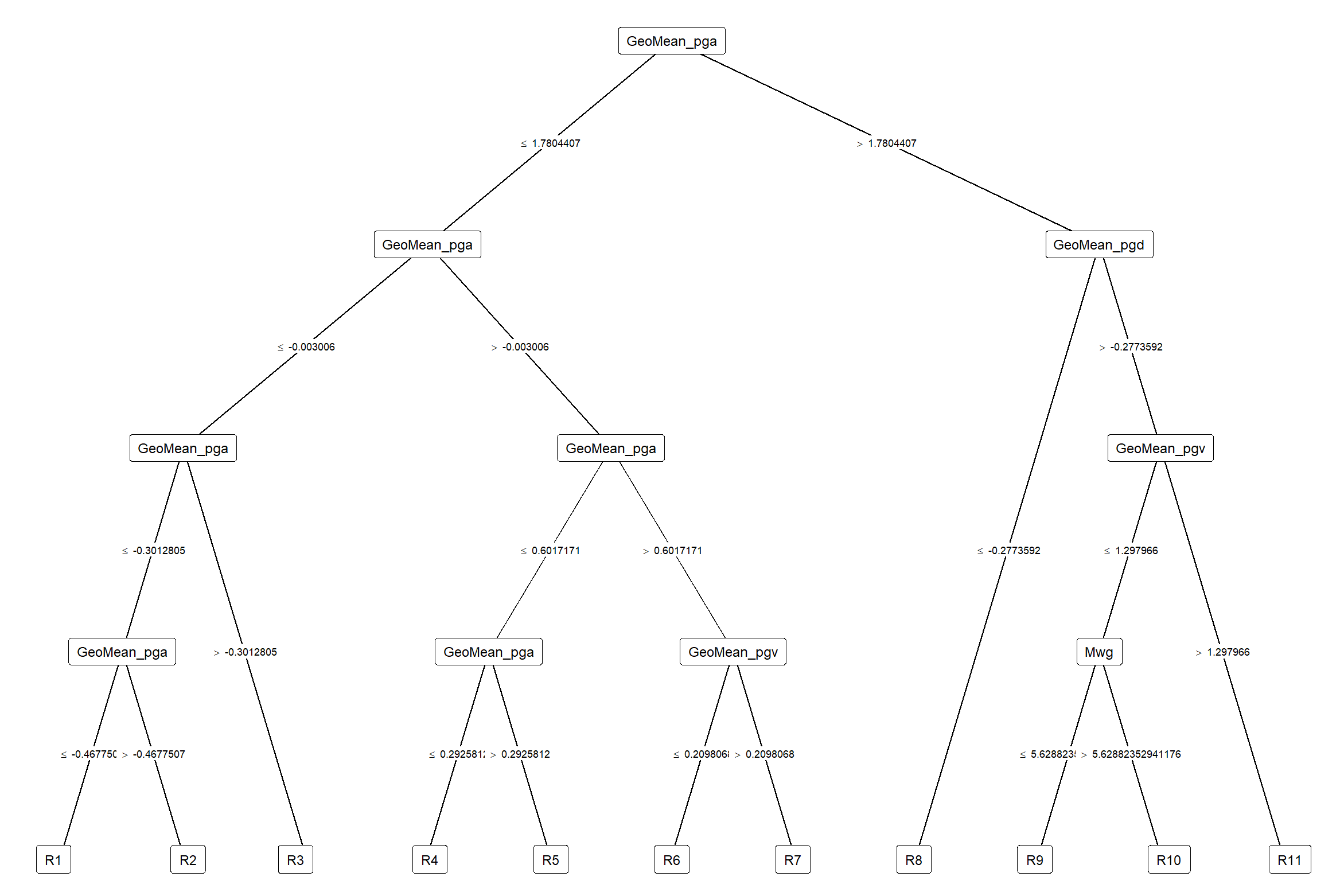
**
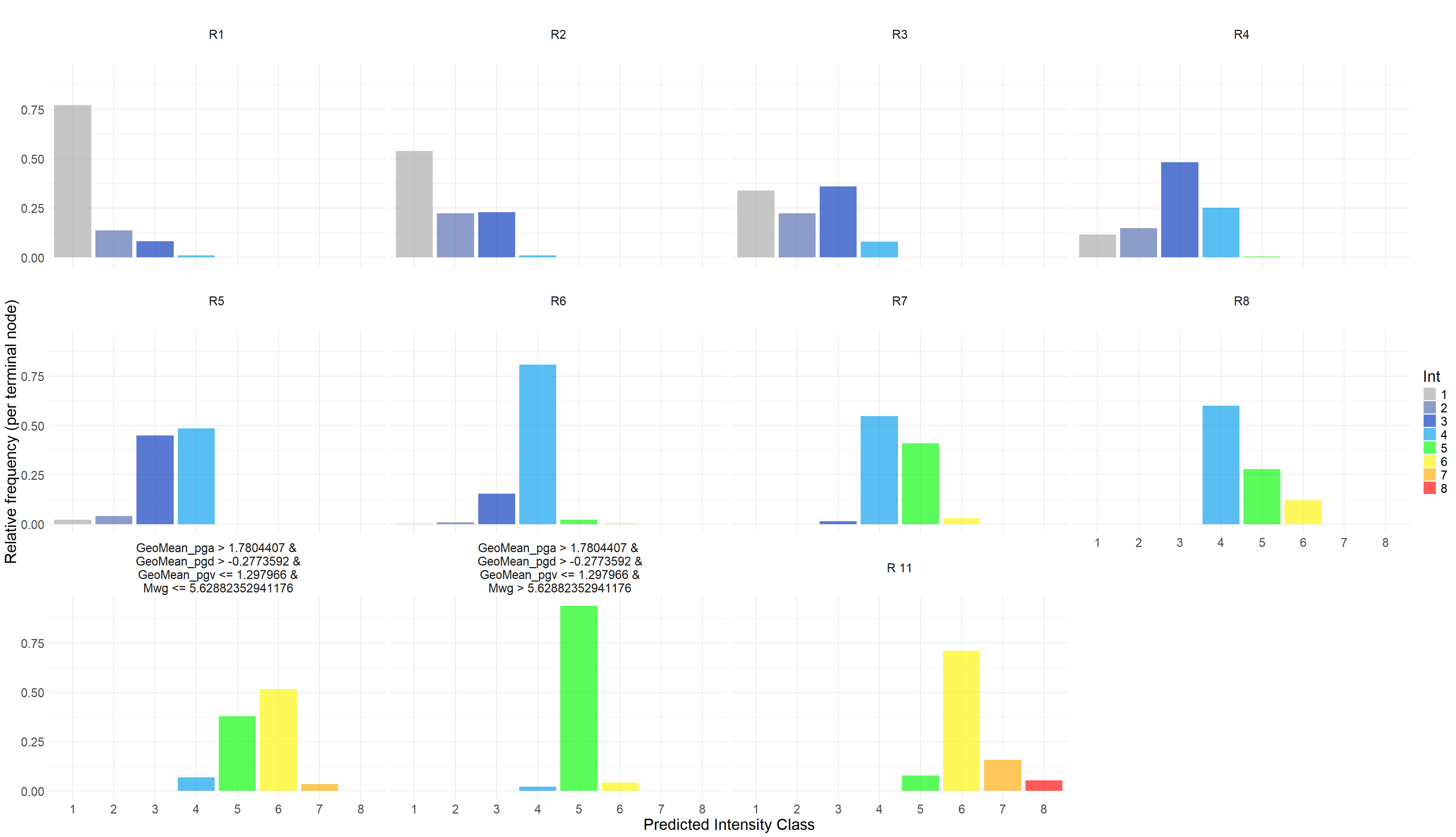
Fig. SI-2-3.** Top: surrogate tree of depth 4 (S4), wherein the rectangles represent the predictor for the split, and the conditions are displayed along the branches. Bottom: distribution of the intensity classes in the eleven terminal nodes of S4 ($R_{1}$, ..., $R_{11}$).

| **Model evaluation metrics** | | | | | |
| --- | --- | --- | --- | --- | --- |
| **Method** | **Acc.** | **Sens.** | **Prec.** | **Spec.** | **F1** |
| RF | 0.977 | 0.755 | 0.826 | 0.99 | 0.789 |
| S3 | 0.971 | 0.500 | 0.979 | 0.999 | 0.662 |

Acc. = accuracy , Sens. = sensitivity, Prec. = precision, Spec. = specificity

**Table SI-2-1.** The test predictive performance indexes for RF and S3 models in the case of “Low” vs “High” binary categories.

We observed no statistically significant differences with respect to the use of standard $M_{w}$ in terms of model evaluation metrics for the adopted Machine Learning models (RF and Surrogate Trees of different depths). Differently, we obtain a new cutoff value in the last split of the surrogate of depth 4 (S4) which changes from 5.9 (when using $M_{w}$) to 5.6 (when using Mwg). This shift in the cutoff value is consistent with the transformation performed on the Mw values. Noteworthy, the other 9 splits (of S4) remain identical.
